# Supplementary material for: The Role of the Social Determinants of Health on Engagement in Physical Activity or Exercise among Adults Living with HIV: A Scoping Review
Source: Int J Environ Res Public Health. 2022 Oct 19;19(20):13528. doi: 10.3390/ijerph192013528 (PMC9602835; doi:10.3390/ijerph192013528)
Supplement: Supplementary file 1 [file ijerph-19-13528-s001.zip › Supplemental File-S1-PHAC-SDOH-Descrpitions-Defintion-FINAL-SUBMITTED-Jul-31-22.pdf]

Supplemental File S1: Public Health Agency of Canada Social Determinants of Health Framework

Social Determinant of Health	Definition / Description
**Biology and Genetic Endowment**	Certain genes can make us more susceptible to certain health conditions.
**Childhood Experiences**	Adequate income, effective parenting and family functioning and supportive community environments play a key role in child development, which has been well connected to health outcomes when they are adults and age.
**Education and Literacy**	Being well educated equates to a better job, higher income, greater health literacy, a wider understanding of the implications of unhealthy behaviour and an increased ability to navigate the health care system, leading to better health care.
**Employment and Working Conditions**	Although there are employment regulations to protect Canadians, there are significant differences in the number of injuries, stress and job security experienced by individuals. Those working blue-collared jobs and earning less than 40 000 are more likely to be injured.
**Healthy Behaviours**	Individual behaviours such as healthy eating, smoking and consuming alcohol can have a significant effect on one's health.
**Access to Health Services**	Access to healthcare is essential to health, it includes medical services but also health promotion and prevention such as vaccinations
**Gender**	Gender plays a key role in power, social and culture and can have an impact on health status.
**Physical Environments**	Affordable, safe and resource rich neighbourhoods are linked to better health outcomes.
**Income and Social Status**	Relationship with income has shown significant differences in disease prevalence and years lost of life. Due to access to resources increasing with income, people of higher income have lower prevalence compared to people with lower incomes.
**Race/Racism**	Racism and race are well connected to stress and accessibility of health services which can take a toll on health status.
**Culture**	Cultural practices can affect other determinants and our health status.
**Social Supports and Coping Skills**	Satisfaction with self and community, problem-solving capabilities and the ability to manage life situations can contribute to better health overall

Reference: Public Health Agency of Canada. Social determinants of health and health inequalities Public Health Agency of Canada; 2019 [Available from: https://www.canada.ca/en/public-health/services/health-promotion/population-health/what-determines-health.html.
