# Supplementary material for: The Role of the Social Determinants of Health on Engagement in Physical Activity or Exercise among Adults Living with HIV: A Scoping Review
Source: Int J Environ Res Public Health. 2022 Oct 19;19(20):13528. doi: 10.3390/ijerph192013528 (PMC9602835; doi:10.3390/ijerph192013528)
Supplement: Supplementary file 1 [file ijerph-19-13528-s001.zip › Supplemental File-S2-Search-Strategy-Example-FINAL-SUBMITTED-Jul-31-22.pdf]

## Supplemental File S2: Scoping Review Search Strategy Example

**Database: Ovid MEDLINE: Epub Ahead of Print, In-Process & Other Non-Indexed Citations, Ovid MEDLINE® Daily and Ovid MEDLINE® <1946-Present>**
**Search Strategy:**

--------------------------------------------------------------------------------

1. exp Residence Characteristics/
2. exp Environment Design/
3. exp Marital status/
4. neighbo?rhood*.mp.
5. residential environment*.mp.
6. rural*.mp.
7. (inner?city or inner? cities).mp.
8. housing instab*.mp.
9. housing unstab*.mp.
10. housing insecur*.mp.
11. housing strain*.mp.
12. housing security.mp.
13. (mortgage* adj3 problem*).mp.
14. foreclosure*.mp.
15. evict*.mp.
16. (housing adj3 los*).mp.
17. (home adj3 repossession*).mp.
18. (home adj3 ownership*).mp.
19. (repossess* adj3 propert*).mp.
20. (mortgage* adj3 delinquen*).mp.
21. (mortgage* adj3 arrear*).mp.
22. (mortgage* adj3 debt*).mp.
23. overcrowd*.mp.
24. (living adj1 (outside or inside or near* or adjacent)).mp.
25. (household* adj2 size*).mp.
26. (marital status or marriage status).mp.
27. (widow* or cohabit* or divorce* or unmarried or single parent* or liv* alone).mp.
28. exp Cultural Deprivation/
29. exp Acculturation/
30. Culture/
31. exp Cross-Cultural Comparison/
32. exp Cultural Characteristics/
33. exp Cultural Diversity/
34. Language/
35. exp "Transients and Migrants"/
36. exp "Emigrants and Immigrants"/
37. exp Minority groups/
38. exp Minority health/
39. exp Prejudice/
40. exp Racism/
41. exp Xenophobia/

42. exp Social Discrimination/
43. exp Race Relations/
44. exp Ethnic Groups/
45. exp Continental Population Groups/
46. exp Refugees/
47. minorit*.mp.
48. (migration* adj3 background*).mp.
49. racial?.mp.
50. racism?.mp.
51. ethnology.mp.
52. race.mp.
53. ethnic*.mp.
54. non? English.mp.
55. language other than.mp.
56. (latino* or latina or latinx*).mp.
57. hispanic*.mp.
58. white?.mp.
59. caucasian*.mp.
60. non?white.mp.
61. exp American Native Continental Ancestry Group/
62. torres strait islander.mp.
63. aboriginal*.mp.
64. american indian*.mp.
65. inuit*.mp.
66. eskimo*.mp.
67. first nation*.mp.
68. indigenous*.mp.
69. english as a second language.mp.
70. english as a 2nd language.mp.
71. foreign language.mp.
72. black?.mp.
73. african american?.mp.
74. immigrant*.mp.
75. immigration*.mp.
76. asian?.mp.
77. metis?.mp.
78. Occupations/
79. Unemployment/
80. occupations.mp.
81. unemploy*.mp.
82. underemploy*.mp.
83. under employ*.mp.
84. exp Sex Factors/
85. exp Gender Identity/
86. exp Women's Health/
87. gender difference*.mp.
88. (sex disparit* or sex difference?).mp.

89. gender identit*.mp.
90. sex role?.mp.
91. wom#n* role?.mp.
92. m#n* role?.mp.
93. gender* role?.mp.
94. servicewom#n?.mp.
95. service wom#n?.mp.
96. servicem#n?.mp.
97. service m#n?.mp.
98. exp Educational status/
99. Education/
100. schooling.mp.
101. education* status.mp.
102. (education* adj2 level?).mp.
103. ((higher or better or worse or less) adj educated).mp.
104. ((higher or better or worse or less) adj level? of education).mp.
105. college educat*.mp.
106. university educat*.mp.
107. Religion/
108. religi*.mp.
109. exp "Social Determinants of Health"/
110. exp Psychosocial Deprivation/
111. exp Sociological Factors/
112. exp Working Poor/
113. exp Hierarchy, Social/
114. disparit*.mp.
115. inequalit*.mp.
116. inequit*.mp.
117. equity?.mp.
118. deprivation?.mp.
119. gini.mp.
120. concentration index?.mp.
121. Socioeconomic Factors/
122. Social Welfare/
123. exp Social Class/
124. exp Poverty/
125. Income/
126. Social class*.mp.
127. social determinant*.mp.
128. social status?.mp.
129. social position?.mp.
130. social background?.mp.
131. social circumstance*.mp.
132. socio-economic*.mp.
133. socioeconomic?.mp.
134. socioeconomic*.mp.
135. sociodemographic*.mp.

136. socio-demographic*.mp.
137. SES.mp.
138. disadvantaged?.mp.
139. impover*.mp.
140. poverty*.mp.
141. low* class?.mp.
142. lowerclass?.mp.
143. under class?.mp.
144. underclass?.mp.
145. economic level?.mp.
146. assets index?.mp.
147. income*.mp.
148. exp Social Stigma/
149. exp Social Capital/
150. Social Control, Informal/
151. exp Social Support/
152. exp Social Environment/
153. exp Trust/
154. Social conditions/
155. exp Social Isolation/
156. exp Social Marginalization/
157. exp Anomie/
158. exp Social Participation/
159. social exclusion*.mp.
160. (social adj (capital or cohes* or organis* or organiz*)).mp.
161. (community? adj3 (cohes* or participa*)).mp.
162. ((neighbourhood? or neighborhood?) adj cohes*).mp.
163. social relationship*.mp.
164. social network*.mp.
165. collective efficacy.mp.
166. civil society?.mp.
167. informal social control?.mp.
168. neighbo*rhood? disorder*.mp.
169. social disorgani?ation.mp.
170. anomie?.mp.
171. social support?.mp.
172. social participation*.mp.
173. trust.mp.
174. emotional support*.mp.
175. psychosocial support*.mp.
176. community capital?.mp.
177. neighbo*rhood cohesi*.mp.
178. social influence?.mp.
179. (soci*context* or soci*-context*).mp.
180. exp Health Status Disparities/
181. Health Services Accessibility/
182. exp Health Equity/

183. health*care disparit*.mp.
184. health care disparit*.mp.
185. health status disparit*.mp.
186. health disparit*.mp.
187. health inequalit*.mp.
188. health inequit*.mp.
189. health equalit*.mp.
190. medically underserved.mp.
191. exp Adverse Childhood Experiences/
192. exp Child Development/
193. exp Child Abuse/
194. exp Child, Abandoned/
195. Child Behavior/
196. exp Child Health/
197. exp Infant Health/
198. Child Welfare/
199. exp Child of Impaired Parents/
200. exp Child, Orphaned/
201. exp Parent-Child Relations/
202. exp Child, Unwanted/
203. Child Health Services/
204. child*.mp.
205. exp Genetics/
206. exp Genetic Markers/
207. ge.fs. [genetics subheading]
208. genetic*.mp.
209. exp Biology/
210. biolog*.mp.
211. Health Behavior/
212. Attitude to health/
213. exp Smoking/
214. exp Tobacco Products/
215. exp "Tobacco Use Disorder"/
216. exp Alcohol Drinking/
217. exp Alcoholic Beverages/
218. exp Alcohol-Related Disorders/
219. (attitud* adj3 health*).mp.
220. (health* adj3 Behavior?).mp.
221. (health* adj3 Behaviour?).mp.
222. smok*.mp.
223. tobacco*.mp.
224. alcohol*.mp.
225. exp Homeless Persons/
226. exp Vulnerable Populations/
227. homeless*.mp.
228. (underprivileg* adj3 (population* or person* or people* or child* or patient*)).mp.
229. (vulnerable* adj3 (population* or person* or people* or child* or patient*)).mp.

230. (marginali* adj3 (population* or person* or people* or child* or patient*)).mp.
231. potential determinant*.mp.
232. significant correlat* of.mp.
233. (independent* correlate* or independent* associat*).mp.
234. variable* associat* with.mp.
235. determinant* of.mp.
236. factor* associated with.mp.
237. identif* determinant*.mp.
238. (more likely or less likely or just as likely).mp.
239. risk factor* for.mp.
240. (significantly related to or significant predictor*).mp.
241. (also adj2 associated with).mp.
242. (at increased risk* or at decreased risk*).mp.
243. association* between.mp.
244. (positively associated or negatively associated).mp.
245. differed by.mp.
246. (were high* amongst or were low* amongst).mp.
247. (inverse relationship with or inversely associated with or inversely related to).mp.
248. reverse association*.mp.
249. differentially affects.mp.
250. (evidence of adj1 link* between).mp.
251. (significant* adj3 likelihood of).mp.
252. protective factor* for.mp.
253. (differ* adj2 according to).mp.
254. (inverse adj2 gradient*).mp.
255. (positive adj2 gradient*).mp.
256. (negative adj2 gradient*).mp.
257. (trend* adj2 across).mp.
258. (relat* to adj3 variable*).mp.
259. (differenc* adj3 explained by).mp.
260. (significan* among or no# significan* among).mp.
261. exp HIV/
262. exp Acquired Immunodeficiency Syndrome/
263. exp HIV Seropositivity/
264. exp HIV Infections/
265. exp HIV Long-Term Survivors/
266. HIV?.mp.
267. AIDS.mp.
268. (human adj3 immunodeficien* adj3 virus*).mp.
269. (acquired adj3 immun* adj3 deficienc* adj3 syndrome*).mp.
270. exp Exercise/
271. exp Exercise Therapy/
272. exp Exercise Movement Techniques/
273. exp Physical Fitness/
274. exp "Physical Education and Training"/
275. exp Sports/
276. Recreation/

277. (exercise* or exercising).mp.
278. (qi gong or qigong or gi gong or gigong).mp.
279. ((tai adj ji) or ((tai or thai) adj chi) or taiji or taijiquan or taichi).mp.
280. walk*.mp.
281. yoga?.mp.
282. (physical* adj (fit or fitness or condition* or education or training or mobility or activit* or exertion or effort or program* or therap*)).mp.
283. gymnastic?.mp.
284. calisthenic?.mp.
285. aerobic?.mp.
286. danc*.mp.
287. (jumping or hopping).mp.
288. muscle strengthening.mp.
289. ((strength or resistance) adj training).mp.
290. (fitness adj training).mp.
291. ((weight? adj2 lifting) or weightlifting or power lifting or weight training).mp.
292. pilates.mp.
293. stretching.mp.
294. plyometric*.mp.
295. (cardio* adj (conditioning or training)).mp.
296. ((physical or motion or movement or recreation or activity) adj therap*).mp.
297. isometric training.mp.
298. climbing.mp.
299. cycling.mp.
300. (bike or biking).mp.
301. (swim or swimming).mp.
302. (training adj (course* or program*)).mp.
303. kinesi?therap*.mp.
304. sport?.mp.
305. ((multimodal or multi-modal or multicomponent or multi-component) adj training).mp.
306. balance training.mp.
307. ((lifestyle or physical) adj3 (course* or program* or module?)).mp.
308. or/1-260 [SDOH set]
309. or/261-269 [HIV/AIDS set]
310. or/270-307 [exercise/physical activity set]
311. exp animals/ not humans.sh.
312. 308 and 309 and 310
313. 312 not 311
314. limit 313 to yr="1996 -Current"
