# Supplementary material for: The Role of the Social Determinants of Health on Engagement in Physical Activity or Exercise among Adults Living with HIV: A Scoping Review
Source: Int J Environ Res Public Health. 2022 Oct 19;19(20):13528. doi: 10.3390/ijerph192013528 (PMC9602835; doi:10.3390/ijerph192013528)
Supplement: Supplementary file 1 [file ijerph-19-13528-s001.zip › Supplemental File-S3-Data-Extraction-Variables-Definitions-SDOH-Exercise-PA-FINAL-SUBMITED-Jul-31-22.pdf]

Supplemental File S3: Characteristics Extracted from Included Articles – Operational Definitions

Characteristic / Variable	Operational Definition
**Article**	Title of the article
**Author(s)**	Authors of the article
**Year of publication**	Year article was published
**Country(s) in which the study was conducted**	Where the study has conducted/where were participants recruited.
**City and country of first author**	City and Country of Lead Author
**Study purpose**	Purpose for conducting the study as indicated by the authors
**Study objectives**	Specific study objectives indicated by authors if different from study purpose
**Does the primary focus of the study assess the relationship of SDOH with PA?**	Yes, No, Don't Know, or Not Clear
**Do the study sub-objectives assess the relationship of SDOH with PA?**	Yes, No, Don't Know, or Not Clear
**Study design**	Cross-Sectional, Cohort, Case-Control, Randomized Controlled Trial, Narrative Review, Systematic Review, Qualitative Study, Intervention Study, etc.
**Type of article**	Primary data collection; review article (narrative review, systematic review); editorial; commentary; secondary data analysis, etc.
**Data collection methods**	Qualitative - focus groups, semi-structured interviews; Quantitative: questionnaires, chart extraction, physical examination, medical records, different PA measurement tools, etc.
**Duration of study**	If a study involved primary data collection - length of follow up [only for longitudinal studies]
**Inclusion criteria of participants**	As reported by study authors
**Exclusion criteria of participants**	As reported by study authors
**Sample size at baseline**	Sample size of participants at baseline
**Sample size at study completion**	Sample size of participants at study completion (if applicable)
**Attrition rate**	(# of participants at baseline- # of participants at end of study) divided by number at baseline.
**Study population**	Refers to details pertaining to characteristics of the study sample included in the article. Example: sex; gender; age; race/ethnicity; employment status; education; housing status; number of participants living with HIV, number of participants taking ART at baseline; concurrent health conditions / comorbidities; number of participants engaged in physical activity at baseline (if reported) and extent of engagement.

Characteristic / Variable	Operational Definition
**Type of outcomes measured**	Types of outcome measures referred to in the article (if any; nature of physical Activity and exercise: type (aerobic, flexibility, resistive and neuromotor / balance), extent of physical activity and exercise includes frequency, time, intensity, and progression).<br>If applicable, primary outcome of interest (and how measured) and when; secondary outcomes of interest (and how measured);<br>How was physical activity or exercise defined in this study; PA or exercise assessment / how measured.
**Intervention (If Applicable)**	Refers to the characteristics of the intervention (if applicable) including frequency; intensity; time; type of intervention; control and intervention group (if applicable), sample size of control and intervention group at baseline and study completion (if applicable)
**Conceptual framework**	Was there any conceptual framework used in the article? (Yes or No); For example, SDOH Framework? If yes, name of framework
**Name of the SDOH reported**	What are the SDOH reported in the article in relation to PA?
**Author's results and conclusions**	Authors' overall conclusion and key findings as stated in the article.
**Author's results and conclusions related to SDOH and PA**	What were the authors results / conclusions related to SDOH and PA?
**Reviews Interpretations**	Reviewer interpretations about this article as it relates to SDOH and physical activity or exercise in the context of HIV. For example: 1) how the included article addresses what is known (or not known) about the relationships between the SDOH and PA or exercise among adults living with HIV; 2) interpretations of any gaps in knowledge pertaining to SDOH and physical activity and exercise for adults living with HIV.

Abbreviations: PA, Physical Activity; SDOH, Social Determinants of Health
