# Supplementary material for: The Role of the Social Determinants of Health on Engagement in Physical Activity or Exercise among Adults Living with HIV: A Scoping Review
Source: Int J Environ Res Public Health. 2022 Oct 19;19(20):13528. doi: 10.3390/ijerph192013528 (PMC9602835; doi:10.3390/ijerph192013528)
Supplement: Supplementary file 1 [file ijerph-19-13528-s001.zip › Supplemental File-S4-Citations-Excluded-Articles-SDOH-Exercise-PA-FINAL-SUBMITTED-Jul-31-22.pdf]

Supplemental File S4: Excluded Articles Classified as Indirect Studies exploring Social Determinants of Health and Physical Activity and Exercise among Adults Living with HIV (n=16)

1. Masters MC, Perez J, Tassiopoulos K, Andrade A, Ellis R, Yang J, Brown TT, Palella FJ , Jr, Erlandson KM. Gait Speed Decline Is Associated with Hemoglobin A1C, Neurocognitive Impairment, and Black Race in Persons with HIV. AIDS Res Hum Retroviruses. 2019 Nov-Dec;35(11-12):1065-1073. doi: 10.1089/AID.2019.0101. Epub 2019 Sep 30. PMID: 31468979; PMCID: PMC6862955
2. McKellar MS, Kuchibhatla MN, Oursler KAK, Crystal S, Akgün KM, Crothers K, Gibert CL, Nieves-Lugo K, Womack J, Tate JP, Fillenbaum GG. Racial Differences in Change in Physical Functioning in Older Male Veterans with HIV. AIDS Res Hum Retroviruses. 2019 Nov/Dec;35(11-12):1034-1043. doi: 10.1089/AID.2018.0296. Epub 2019 Jun 18. PMID: 30963773; PMCID: PMC6862951.
3. Martin K, Naclerio F, Karsten B, Vera JH. Physical activity and quality of life in people living with HIV. AIDS Care. 2019 May;31(5):589-598. doi: 10.1080/09540121.2019.1576848. Epub 2019 Feb 2. PMID: 30712360.
4. Zhang J, O'Leary A, Jemmott JB 3rd, Icard LD, Rutledge SE. Syndemic conditions predict lower levels of physical activity among African American men who have sex with men: A prospective survey study. PLoS One. 2019 Mar 13;14(3):e0213439. doi: 10.1371/journal.pone.0213439. PMID: 30865694; PMCID: PMC6415907.
5. Mabweazara, S., Leach, L. & Ley, C. Development of a context-sensitive physical activity intervention for persons living with HIV and AIDS of low socioeconomic status using the behaviour change wheel. BMC Public Health 19, 774 (2019). https://doi.org/10.1186/s12889-019-7091-8
6. Li A, McCabe T, Silverstein E, Dragan S, Salbach NM, Zobeiry M, Beldick S, Godi C, O'Brien KK. Community-Based Exercise in the Context of HIV: Factors to Consider when Developing and Implementing Community-Based Exercise Programs for People Living with HIV. J Int Assoc Provid AIDS Care. 2017 May/Jun;16(3):267-275. doi: 10.1177/2325957416686836. Epub 2017 Jan 11. PMID: 28074681.
7. Montgomery CA, Henning KJ, Kantarzhi SR, Kideckel TB, Yang CF, O'Brien KK. Experiences participating in a community-based exercise programme from the perspective of people living with HIV: a qualitative study. BMJ Open. 2017 Apr 4;7(4):e015861. doi: 10.1136/bmjopen-2017-015861. PMID: 28377397; PMCID: PMC5387963.
8. Malete L, Tladi DM, Etnier JL, Makhanda J, Anabwani GM (2019) Examining psychosocial correlates of physical activity and sedentary behavior in youth with and without HIV. PLOS ONE 14(12): e0225890. https://doi.org/10.1371/journal.pone.0225890
9. Dufour CA, Marquine MJ, Fazeli PL, et al. Physical exercise is associated with less neurocognitive impairment among HIV-infected adults. J Neurovirol. 2013;19(5):410-417. doi:10.1007/s13365-013-0184-8
10. Iorindo AA, de Oliveira Latorre Mdo R, Jaime PC, Segurado AA. Leisure time physical activity prevents accumulation of central fat in HIV/AIDS subjects on highly active antiretroviral therapy. Int J STD AIDS. 2007 Oct;18(10):692-6. doi: 10.1258/095646207782193795. Erratum in: Int J STD AIDS. 2010 Jun;21(6):452. PMID: 17990379.
11. Montoya JL, Wing D, Knight A, Moore DJ, Henry BL. Development of an mHealth Intervention (iSTEP) to Promote Physical Activity among People Living with HIV. J Int Assoc Provid AIDS Care. 2015 Nov-

Dec;14(6):471-5. doi: 10.1177/2325957415601505. Epub 2015 Aug 25. PMID: 26307212; PMCID: PMC4654667.

12. Webel AR, Willig AL, Liu W, Sattar A, Boswell S, Crane HM, Hunt P, Kitahata M, Matthews WC, Saag MS, Lederman MM, Rodriguez B. Physical Activity Intensity is Associated with Symptom Distress in the CNICS Cohort. AIDS Behav. 2019 Mar;23(3):627-635. doi: 10.1007/s10461-018-2319-7. PMID: 30368620; PMCID: PMC6408232.
13. Chow, F. C., Makanjuola, A., Wu, K., Berzins, B., Kim, K-Y. A., Ogunniyi, A., Ellis, R. J., Robertson, K., Tassiopoulos, K., & Taiwo, B. O. (2019). Physical Activity Is Associated with Lower Odds of Cognitive Impairment in Women but Not Men Living with Human Immunodeficiency Virus Infection. Journal of Infectious Diseases, 219(2), 264-274. https://doi.org/10.1093/infdis/jiy503
14. Munro S, Dinatale E, Hartley S, St Jacques M, Oursler KA. Barriers and Health Beliefs Related to Weight Management Among Veterans With Human Immunodeficiency Virus. Mil Med. 2017 Jan;182(1):e1596-e1602. doi: 10.7205/MILMED-D-16-00086. PMID: 28051979.
15. Ramírez-Marrero FA, Rivera-Brown AM, Nazario CM, Rodríguez-Orengo JF, Smit E, Smith BA. Self-reported physical activity in Hispanic adults living with HIV: comparison with accelerometer and pedometer. J Assoc Nurses AIDS Care. 2008 Jul-Aug;19(4):283-94. doi: 10.1016/j.jana.2008.04.003. PMID: 18598903.
16. Smit E, Crespo CJ, Semba RD, Jaworowicz D, Vlahov D, Ricketts EP, Ramirez-Marrero FA, Tang AM. Physical activity in a cohort of HIV-positive and HIV-negative injection drug users. AIDS Care. 2006 Nov;18(8):1040-5. doi: 10.1080/09540120600580926. PMID: 17012097.
